# Development and Early Implementation of a Parent Peer Programme for Rural and Remote Communities: An Evaluation of Feasibility and Acceptability

**DOI:** 10.1111/hex.70967

**Published:** 2026-11-10

**Authors:** Kerri M. Gillespie, Megan McClenaghan, Nataya Branjerdporn, Simon Jockel, Grace Branjerdporn

**Affiliations:** ^1^ Mater Research Institute – University of Queensland St Lucia Queensland Australia; ^2^ Faculty of Medicine University of Queensland St Lucia Queensland Australia; ^3^ School of Clinical Sciences, Translational Research Institute Queensland University of Technology Woolloongabba Queensland Australia; ^4^ Catherine's House for Mothers, Babies and Families, Mater Misericordiae Ltd South Brisbane Queensland Australia; ^5^ Outback Futures Woolloongabba Queensland Australia

**Keywords:** community health services, lived experience, parenting, peer support, Peer work, remote, rural mental health

## Abstract

**Objective:**

The aim of this study was to evaluate the acceptability and feasibility of a Parent Peer support programme in rural and remote Queensland to support parents and improve mental wellbeing, and to describe programme co‐design and implementation.

**Setting:**

Central Queensland.

**Participants:**

Parent Peers and project stakeholders involved in the co‐design, development, and implementation of a rural and remote Parent Peer support programme.

**Design:**

A mixed‐methods study using online surveys and interviews explored the co‐design, implementation, and perceptions of a Peer Support programme for parents in four rural and remote Queensland communities and a Parent Peer training programme. Seven Parent Peers and 13 stakeholders provided perspectives on the co‐design process and the implementation of the Parent Peer programme. Parent Peers also provided feedback on the Peer Support training modules. Descriptive statistics and thematic and summative content analysis were used to analyse and describe quantitative and qualitative data.

**Results:**

Two themes were identified, including ‘Connection and Collaboration’, which identified the collaborative, co‑design process as crucial for the successful development, implementation, and sustainability of the programme; and ‘An Evolving Role’, which acknowledged the importance of the evolving and adaptable nature of the role to ensure tailored and responsive strategies for individual communities. Training was acceptable and engaging for Parent Peers, providing them with valuable education and support as they began their role.

**Conclusion:**

The co‐designed Parent Peer programme was feasible, acceptable, and strongly supported by participants. The Parent Peer role needed to be flexible and adaptable to meet changing and unique community needs. The Parent Peer model could be widely implemented in rural and remote communities across Australia to improve parent support and wellbeing.

**Patient or Public Contribution:**

An iterative consultative co‐design process was systematically followed throughout. Organisational healthcare professionals,individuals with lived experience, parents, early education specialists, and community stakeholders were actively involved in the design, development, and implementation of the Parent Peer project.

## 1 Introduction

New parents face challenges during the transition into parenthood, resulting from significant changes to their identity, responsibilities, and expectations, as well as an increased susceptibility to stress, fatigue, and social vulnerability [1, 2, 3]. Support for parents throughout early parenting phases is critical, as this is a crucial time for parent‐child attachment formation, infant development, and a period of high vulnerability for parental well‐being [1, 2]. New parents within rural and remote communities experience additional difficulties due to their geographic isolation, such as limited access to services, exacerbated social vulnerability, and social stigma [4]. In Australia, remoteness is formally classified according to relative geographic access to services, and ranges from RA1 (Major city) to RA5 (Very Remote) [5]. To access appropriate support services, rural parents often must travel long distances, and telehealth alternatives frequently prove unsatisfactory because of internet connectivity issues and approaches that do not adequately reflect local circumstances and preferences [4, 6].

Sensitivity and responsiveness to local contexts are important considerations when developing and implementing programmes for rural and remote communities. These approaches acknowledge the broader social and cultural characteristics of rural and remote communities. Geographical barriers and limited resources mean rural and remote communities often differ significantly from metropolitan settings in ways of life, access to childcare and childhood education, family and community relationships, daily routines, and healthcare needs and preferences [7, 8, 9]. These differences can shape parents' experiences of the transition to parenthood, parenting practices and expectations, parental roles, and engagement with healthcare and community services [10, 11]. Consequently, parenting support programmes need to be responsive to the distinct circumstances, priorities, and preferences of the communities in which they are delivered.

Locally responsive and accessible parenting support services are needed for geographically disparate parents within rural and remote communities. Early parenting interventions have been shown to improve parenting outcomes, promote empowerment, and support ongoing child development [2, 12]. The structured social relationship provided by peer support poses a promising solution to the gap in appropriate and accessible formal support services within rural perinatal healthcare [13, 14]. Peer support workers have a lived experience of parenting, and are employed on a paid or volunteer basis to provide social‐emotional and practical support to parents through a lens of shared experiences and understanding [15, 16]. Peer support has demonstrated efficacy within both perinatal mental health and parenting fields [14, 17, 18]. Parents who have accessed peer support have noted improvements to their parenting behaviour and competence, social‐emotional wellbeing, their relationship with their child, and child‐related outcomes [13, 18].

Community acceptability of peer support programmes is facilitated by their successful integration into existing healthcare systems [16], which subsequently improves parents' access to high‐quality, evidence‐based healthcare [14]. Peer support models allow remote service delivery, which is why they appear well suited to supporting parents in rural communities [4]. Despite their apparent promise, recruitment and sustainability are often challenging in rural and remote regions because of a smaller workforce pool, a transient workforce, broader skill requirements, limited funding, geographic isolation, and limited access to infrastructure, supervision, and training [1, 12, 19, 20].

Literature supporting the benefits of peer work emphasises the importance of appropriate peer training, supervision, and acknowledgement [14, 15]. When training remote peer workers, online learning methods combat geographic barriers, provide a cost‐effective mode of health‐related education delivery, and facilitate ease of training accessibility and scalability [21]. Digital training delivery additionally allows for flexible, self‐directed learning that is well‐suited to remote education [21, 22]. However, online training, particularly in rural and remote areas, can be impacted by connectivity issues, digital access, and deficient infrastructure, may negatively affect concentration and communication, and leaves fewer opportunities to practice skills in more realistic settings [23, 24]. Hybrid approaches that combine self‐directed online training with facilitated discussion may therefore be superior for preparing peer workers in rural and remote settings. When aiming to address a critical social need, such as remote early‐parenting support, it is especially crucial to evaluate the design, implementation, and outcomes of peer worker training within such programmes [21].

The current Parent Peer programme was developed to support parents of young children living in rural and remote communities who may experience social isolation and limited access to healthcare and psychosocial supports. A locally embedded Parent Peer programme was hypothesised to address these issues and improve parental wellbeing by providing local, accessible, informal support to facilitate social connection, parent education, and link parents to appropriate services.

This study aimed to evaluate the acceptability and feasibility of the Parent Peer programme, including the initial development of the programme and associated training package through co‐design, and subsequent programme implementation in rural and remote Queensland. The study addressed four main questions: 1) What were Parent Peers' perceptions of the acceptability, usefulness, and relevance of the training; 2) How could the training be improved; 3) How did Parent Peers and stakeholders perceive the Parent Peer role and future directions of the programme; and 4) What factors and characteristics are associated with successful programme implementation, acceptability, and sustainability?

## 2 Study Background

### 2.1 Co‐Design

All aspects of the programme were designed and developed using a co‐design process. This process entailed the creation of an expert panel comprising individuals with lived experience, parents, early education specialists, community stakeholders, researchers, and clinicians (including a psychologist and general practitioner). The panel met quarterly with healthcare professionals from OBF to develop and refine the programme and the Parent Peer training. The programme was initially anticipated to deliver mental health support to parents at risk of mental illness. However, feedback from the panel suggested that the focus on mental health may alienate members of the community. It was decided that Parent Peers would concentrate on addressing isolation and parenting challenges, connecting parents with appropriate resources when necessary, and maintaining an open approach to address the emerging needs of parents in the community.

Parent Peers were not required to have any formal qualifications or prior training, making the provision of structured training crucial. To address this need, a series of tailored learning modules was developed using the co‐design process. The topics and structure of the modules were based on suggestions from the expert panel. Two consultants reviewed an initial draft, providing a First Nations perspective and a ‘bush’ perspective, and their feedback was integrated into the content. The four topics created based on this consultation process were: Peer Support; Peer Listening; Peer Storytelling; and Peer Resilience.

### 2.2 Outback Futures

Outback Futures (OBF) is a not‐for‐profit organisation based in Central Queensland, Australia, with the ethos of empowering rural and remote communities to improve health outcomes [25]. OBF focuses simultaneously on primary and secondary prevention. Their Community Facilitation Model enables them to work with communities to identify their most significant challenges and co‐design a response to address these. They seek to equip rural and remote communities with the confidence and tools to identify and address their own mental health and wellbeing issues. OBF provides allied health services for children and adults of all ages currently experiencing mild to moderate mental health conditions, or who are at risk of future mental illness. OBF currently supports shires within the Central West region (Longreach, Winton, Barcaldine, Blackall‐Tambo, Barcoo) and North‐West region (Cloncurry, Richmond, Flinders, McKinlay). These areas are considered ‘Very Remote Australia’ according to the Australian Bureau of Statistics Remoteness Area classifications (or Remoteness Area Category 5) [5]. This category reflects the highest level of geographic isolation with the least access to goods and services or large population centres.

### 2.3 The Parent Peer Programme

In response to the service gap for rural parents, OBF created the Parent Peer programme to train and employ rural peer support workers (named Parent Peers) to aid rural parents in remote Queensland communities. Online training modules were created to educate Parent Peers (parents with a lived experience of rural parenting) to provide informal support to other rural parents. OBF hired Parent Peers from each remote Queensland community through advertisements and word of mouth. To be eligible for the position, people must:

- currently live in a rural Queensland community;
- have a lived experience of the early parenting stages within a rural Queensland community;
- not have been born in the rural Queensland community;
- be well‐connected and respected within their rural Queensland community; and
- be willing to take part in the Parent Peer project.

Seven women were employed over a 2‐year period to work as Parent Peers in casual positions for approximately 8 h a week. The Parent Peer role aimed to reach out to parents in the community who may require social support. The position primarily entailed attending playgroups in their local community and facilitating community connections through that. Parent Peers were placed within community groups focused on parents and young children in town centres, improving access for community members and reducing travel requirements for interested parents. Other activities (liaising with healthcare professionals, attending or forming new parent groups, one‐on‐one visits with parents who may be experiencing social isolation or parenting challenges) were performed ad hoc and were contingent on the needs of each community. The Parent Peer role was expected to evolve over time, as Parent Peers developed relationships with parents, connected with local healthcare professionals and resources, and individual community and parent requirements emerged.

### 2.4 Training Modules

The Parent Peer training package was developed using Articulate Rise 360 Articulate Global [26], an interactive online platform that supports flexible, self‐paced learning. The package consisted of four key modules, as described in Table 1. Each module comprised several interactive elements to enhance the learner's experience: animated educational videos; recorded audio and case study examples; question‐and‐answer or word‐matching activities; and relevant images or infographics with interactive information tabs. Links to relevant resources and educational tools were also included to inform participants about the principles of peer support, equipping them to engage in their local communities as Parent Peers, working alongside rural and remote parents. The course was supported by a printable digital workbook, and online group learning components. Completion of the eLearning modules was self‐directed but scheduled so that modules were completed within a 1‐week period. Following completion of each of the four modules, 60‐min debriefing sessions were conducted with the Parent Peer Coordinator and other Parent Peers to consolidate the content and discuss learnings. The Parent Peer Coordinator facilitated the sessions via Zoom. Once completed, a certificate of achievement was generated for each Parent Peer.

**Table 1:** Online module and lesson topics.

Module	Lessons
Module 1: Peer Support	Lesson 1: Introduction to Parent Peer support
	Lesson 2: What does a Parent Peer do?
	Lesson 3: Parent Peer support in action
	Lesson 4: Possible concerns and important considerations
	Lesson 5: Skill building and guidance in this course
	Lesson 6: Summary
Module 2: Peer Listening	Lesson 1: Introduction to peer listening
	Lesson 2: What is peer listening?
	Lesson 3: Practicing active listening ‐ tools, and techniques
	Lesson 4: Types of ineffective listening
	Lesson 5: Summary
Module 3: Peer Storytelling	Lesson 1: Introduction to peer storytelling
	Lesson 2: Tools, purposes, and techniques
	Lesson 3: Your story
	Lesson 4: Summary
Module 4: Peer Resilience	Lesson 1: Introduction to peer resilience
	Lesson 2: Peer courage in conflict
	Lesson 3: Peer self‐care
	Lesson 4: Peer crisis support
	Lesson 5: Summary

## 3 Methods

### 3.1 Study Design

This study used a mixed‐methods approach to evaluate the acceptability and feasibility of the Parent Peer programme in rural and remote Queensland. The evaluation comprised pre‐ and post‐training online surveys to assess Parent Peers' perceptions of the training, including its relevance, acceptability, usefulness, and areas for improvement; and semi‐structured interviews with project stakeholders who engaged in the co‐design and initial implementation of the Parent Peer programme, and Parent Peers following completion of their training. Interviews explored stakeholders' perspectives on the co‐design and implementation process, as well as Parent Peers' experiences of training, expectations and experiences of their role, and perceptions of programme implementation and future sustainability. A mixed‐methods approach using surveys and interviews was selected to provide additional, complementary evidence and enable a more detailed exploration of the Parent Peer programme. Interviews were used to more thoroughly explore the topics assessed quantitatively in the survey.

### 3.2 Ethical Statement

The study was approved by the Mater Hospital Human Research Ethics Committee (ID: HREC/MML/103634). All participants provided written informed consent before taking part in any part of the research study. Parent Peers were emailed a link to the online participant information and consent form. Those who indicated consent by checking ‘I consent’ were directed to the initial pre‐intervention survey, embedded within their first training module. Stakeholders and Parent Peers who agreed to participate in research interviews completed separate online consent forms via the platform, RedCap, prior to completing their interview.

### 3.3 Participants and Recruitment

Participants were recruited via purposive sampling methods to include all Parent Peers and project stakeholders (clinicians and volunteers from a rural allied healthcare service and perinatal peer workers from Mater Hospital, one of Queensland's largest maternity hospitals).

### 3.4 Procedure

Parent Peers completed 4 weeks of training and onboarding before commencing their roles. Training commenced in February 2024 and comprised 1 week of training and onboarding to the OBF organisation, culture, communications, policies, and processes. The final 3 weeks contained the Parent Peer training online modules and debriefing sessions. Pre‐ and post‐surveys were embedded into the online module using the SCORM Cloud LMS platform. Parent Peers were contacted to take part in an interview after they completed the training. Parent Peers were also sent a follow‐up survey at 3 months to evaluate the efficacy of the Parent Peer training process and the initial stages of role implementation. Stakeholders could complete interviews at any time after Parent Peers began their roles in the community.

### 3.5 Data Collection

The semi‐structured interview guide was developed collaboratively by the research team to address the study research questions. The lead author collected data through semi‐structured interviews via videoconferencing software in 2024. Interview questions were devised to investigate participant perceptions of the conception, design, implementation, efficacy, and suggestions or opinions relating to the training modules and the programme development and implementation. Interviews were between nine and 49 min in length (Mean = 30, SD = 8.4) and were recorded and transcribed. Transcripts were manually checked against the interview audio for accuracy. The transcripts were then de‐identified prior to analysis. Data were securely stored on a password‐protected drive accessible only to the research team. Anonymised interviews were returned to participants so they could check that the content reflected their thoughts and opinions. No participants requested changes or provided additional information.

The Parent Peer survey was a bespoke measure developed by the research team specifically for this study. Questions were devised to reflect the aims of the study and to assess Parent Peers' experiences of the training and its impact on their knowledge, skill, self‐reflection, and understanding of the role. Items around satisfaction with the training workbook were also included. Parent Peers completed these surveys online before and immediately after the training.

### 3.6 Author Reflexivity

To maintain consistency, all interviews were conducted by one female research team member KG. At the time of publication, this team member held the position of Senior Research Officer, with a PhD in Natural and Physical Sciences and 18 years of experience in qualitative and quantitative research focused on mental health, nutrition, and chronic disease. This researcher also conducted data analysis. To minimise the risk of bias, all interviews were analysed independently by two members of the research team MM following the principles of thematic content analysis [27]. The other researcher conducting qualitative analysis was a female occupational therapy research clinician with experience in maternity care in Australia. Together, the researchers brought individual and complementary disciplinary perspectives, enhancing the credibility of the findings and the depth of interpretation. Reflexivity was maintained throughout the process by documenting all analytic decisions and discussing any divergent interpretations.

### 3.7 Data Analysis

Quantitative survey data were analysed using the Statistical Package for the Social Sciences (SPSS, version 31.0). Data were analysed descriptively using means and standard deviations or frequencies and percentages. Due to the small sample size and the descriptive aims of the study, no inferential statistics were conducted.

Interviews were analysed using a qualitative descriptive approach to provide a detailed account of participants' experiences and perspectives while remaining close to the data [28]. Data were analysed inductively using the six‐step thematic analysis framework developed by Braun and Clarke [27]. Interview transcripts were read through to identify keywords and phrases to inform the creation of codes, categories, and themes. Interviews were read and re‐read iteratively to identify, refine, confirm, and name occurring themes, which were managed in an Excel spreadsheet. Themes identified by both independent researchers were compared and consolidated until consensus was reached regarding key themes. Where differences in coding or interpretation arose, resolution was achieved through discussion with both researchers. If consensus could not be reached, a third researcher was consulted. Relevant quotes were selected to represent study findings.

Open‐ended survey questions relating to Parent Peers' experiences of the training, feedback, and suggestions for improvement were analysed using summative content analysis [29]. Responses were read iteratively, and key words and phrases were identified and grouped according to shared meaning. The frequency of these words and phrases was then counted to determine their importance.

## 4 Results

### 4.1 Demographics

This study included 20 participants: active Parent Peers (*n* = 7), Parent Peer supervisors (n = 2), Outback Futures staff members (n = 5), advisory group members (n = 4), and researchers (n = 2). Parent Peers were all women with professional backgrounds in education, except one who previously worked in administration (see Table 2). Advisory group members included a peer support worker, a youth worker, a general practitioner, and a dietitian. Outback Futures staff were allied health professionals, a teacher, and a grazier. The researchers involved in the study included an occupational therapist and a psychologist, both with expertise in perinatal mental health. Parent Peers reported spending an average of 6.2 h reviewing the module (between 2.5 and 8.5 h), equating to approximately 1.5 h per module.

**Table 2:** Participant project roles and employment (*n* = 20).

Project role	*N* (%)	Employment background	*N* (%)
Parent peer	7 (35)	Early childhood/primary education	8 (40)
Outback futures team member	5 (25)	Peer support worker	3 (15)
Advisory group member	4 (20)	Occupational Therapist	2 (10)
Research team member	2 (10)	Psychologist	2 (10)
Parent peer supervisor	2 (10)	Speech pathologist	1 (5)
		GP	1 (5)
		Dietician	1 (5)
		Grazier	1 (5)
		Administration support	1 (5)

### 4.2 Qualitative Findings

Qualitative data from interviews with Parent Peers and stakeholders, and from open‐ended questions in Parent Peer surveys, are reported together. These data present opinions and suggestions regarding the development, training, and implementation of the Parent Peer programme. Two key themes were generated from these data: ‘Connection and Collaboration’, which outlines the importance of co‐design and collaboration with OBF and external organisations; and ‘An Evolving Role’, which discusses the importance of the evolving nature of the role itself. Parent Peer feedback from the training and online modules is provided separately.

#### 4.2.1 Theme 1: Connection and Collaboration

Theme one was derived from the interviews with stakeholders involved in the development and co‐design of the Parent Peer programme, including OBF staff, researchers, and advisory group members, and captures their perspectives on the factors that hindered or assisted with developing the programme. Collaboration and connection between OBF, research experts, child health and education specialists, peer workers, and community stakeholders were seen as crucial to the development and implementation of the programme. Partnerships between organisations were seen to benefit training development and delivery, programme design, and implementation by bringing together complementary expertise and backgrounds in research, clinical care, lived experience, and local community knowledge, allowing different stakeholders to contribute expertise that would not have been possible for a single organisation. This collaboration provided support for individual roles and improved efficiency. For example, a metropolitan hospital research team led the research aspects of the study, allowing the Parent Peers and OBF staff to focus on clinical and supportive roles, maintaining their community rapport.

It is really good having… Mater as the research and training type of role and then Outback Futures as the service delivery, and we also provide supervision, so that perinatal mental health expertise.

(Researcher)

The inclusion of lived experience consultants and topic experts is a critical component of the co‐design process. Advisory group members with lived, community, and professional experience provided valuable insight and contributions to the design, refinement, and ongoing progress of the Parent Peer programme. Those with rural community experience provided contextual knowledge of what would be acceptable and feasible. This included developing the criteria for hiring Parent Peers.

…using that consultation from, you know, other professionals and people in communities was absolutely vital to make something that would be effective in communities. I think without that, that input, yeah, we could have floundered.

(OBF staff)

Community stakeholder consultation and collaboration were particularly important to ensure the programme was fit for purpose and met the community's needs.

…we could take it to the Community before implementing it and they could give us … really honest feedback… And the feedback has been really positive, and I think sort of that consultation process really contributed to that.

(OBF staff)

Connection and collaboration between partners were seen as an essential element of the programme. From the perspective of those involved in designing, developing, and implementing the Parent Peer programme, the involvement of individuals with professional, community, research, and lived experience was crucial for a successful, responsive, and appropriately delivered programme that addressed individual community and parent needs. Organisations and individuals were able to work to their strengths, support one another, and collaboratively deliver a product that was fit for purpose and beneficial to the communities involved.

#### 4.2.2 Theme 2: An Evolving Role

This theme reflected the perspectives of both Parent Peers and stakeholders. Parent Peers were not present for the development of the programme but provided insight into the role, role requirements, barriers, enablers, and changes required early in the project's implementation. Participants described how the role necessarily evolved once Parent Peers had begun working in their individual communities. The Parent Peer role was described as difficult to define and was perceived as requiring flexibility or fluidity, especially in the early phases of the project.

…that's part of the evolutionary process with the role too, is just working out, … what that will look like and what the needs are.

(Researcher)

Parent Peers expressed initial uncertainty around the requirements and expectations of the role. All who provided their perspectives of the role described the need to leave room for flexibility and adaptability, especially with the programme still being in its infancy. The programme and the role would evolve as Parent Peers identified parent needs, barriers to service delivery, available resources and opportunities, and the requirements of their individual communities.

…it's a really hard role to define I think because it's so fluid and … it will look different in every town. And because it's a pilot programme, it will sort of evolve and it might change.

(OBF staff)

As Parent Peers became more established in their roles, it was anticipated that they would develop their own strategies for supporting parents in response to locally identified needs, existing services, and available community support. Early examples indicated that some Parent Peers had begun to look for local opportunities to increase their reach or provide greater support to parents. A peer supervisor described how one Parent Peer had ‘started another group to meet the needs of people with younger babies just to help them connect in with their community to form friendships…’. This demonstrated how the role could evolve beyond initially planned activities as Parent Peers identified gaps in community support or opportunities with local organisations and developed locally appropriate activities and responses. As the programme was still in its early stages, further examples of how Parent Peers adapted and expanded their roles are expected to emerge as they gain more experience. This will be explored in subsequent longitudinal interviews.

As the Parent Peer role may develop and evolve, participants recognised that training would also need to be responsive and adaptable. Initial training was based upon community and expert opinions; however, it could not anticipate all situations that Parent Peers may encounter. It was also not developed with one specific community in mind and therefore lacks specificity to address individual locations.

I wonder if their role will grow a little bit … they might need a bit of extra training on how to support parents with special needs kids and things

(OBF staff)

When asked about the importance of the role, all Parent Peers felt that the role was valuable for the community. They felt the purpose of the role was to provide a consistent, approachable presence in the community, engage with parents, implement new activities or events based on need, and follow up with parents who may be experiencing difficulties and could benefit from additional support.

…it can be hard… for parents to sort of enter into groups, I suppose, so just having somebody as a connector I think would be very beneficial.

(Parent Peer)

Overall, the Parent Peer role was described as connecting, empowering, supporting, advocating, and educating rural parents within their local communities. However, individual differences between communities and Parent Peers may lead to variations in roles.

It's different for each region too… each place is gonna have different needs.

(OBF staff)

Not only was flexibility necessary during project development, but it was described as a strength that would drive ongoing adaptability and sustainability. Participants felt that the future direction of the programme should be guided by the community to ensure the programme is accepted, contextually appropriate, and responsive to local needs and interests without being unduly influenced by metropolitan‐based programme developers,

…I think it's better because … the community's driving what they need. And we're not just doing what we think from a metropolitan city point of view, we think would help. It's actually, what do these people in community or out West or in the bush want?

(Researcher)

Adaptability was also understood as a necessary, ongoing feature of the programme, as participants anticipated that the needs of these often‐transient communities would evolve and that unexpected changes may appear over time. Stakeholders also spoke of the need for ongoing reflexivity when working in rural and remote settings.

…acknowledging the fact that this is a rural and remote setting and that there may need to be some, you know, some reflections and some tweaks that need to take place as a result of the unique environment that they're in.

(Peer Worker)

Parent Peers and stakeholders consistently identified similar core functions and benefits of the Parent Peer programme: connecting, empowering, supporting, advocating, and educating parents. They also recognised that the role requirements and activities needed to fulfil these goals would differ across communities and over time. Both Parent Peers and those involved in programme development reported adaptability and flexibility as among the most important characteristics of the Parent Peer role.

### 4.3 Parent Peer Feedback on Training

#### 4.3.1 Survey Findings

Satisfaction surveys completed by Parent Peers after completing the online training modules suggest they were satisfied with the training and believed the modules had helped them develop the skills to be a Parent Peer. Mean scores ranged from 4 to 4.3 out of a possible 5 (see Table 3). The highest‐rated components were the helpfulness of the module, the organisation or flow of the modules, and the fact that the modules encouraged self‐reflection. While overall ratings were high, one Parent Peer scored a 1 or 2 on all satisfaction items, reflecting a rating of disagree or strongly disagree. This participant also noted issues accessing the modules, which may have impacted her ability to engage with them. The workbook was well‐received by all participants. Parent Peers reported consistently high ratings for the workbook's ease of use, usefulness, and capacity to encourage reflection. All participants reported a significantly higher understanding of what the Parent Peer role involved after completing training.

**Table 3:** Survey responses.

Survey item	Mean (SD)	Range
**How long did you spend looking through the online training modules, approximately (in hours)?**	6.2 (1.9)	2.5–8.5
**Satisfaction with training** a		
Overall, I gained the knowledge or skills that I needed to be a successful Parent Peer	4.1 (1.5)	1–5
Overall, I found the online modules helpful	4.3 (1.1)	2–5
The modules were organised and flowed in a logical sequence	4.3 (1.1)	2–5
The modules improved my knowledge (and helped me learn things I didn't know before)	4.1 (1.5)	1–5
The modules were easy to access and use	4 (1.2)	2–5
The modules encouraged me to reflect upon my own biases and other things that may impact my ability to be a successful Parent Peer	4.3 (1.5)	2–5
I found the modules engaging	4.1 (1.5)	1 ‐ 5
**Satisfaction with the workbook** b		
The workbook was easy to use and follow	4.9 (0.4)	4–5
The workbook was useful	4.9 (0.4)	4–5
The workbook helped me reflect on my knowledge and my role as a Parent Peer	4.9 (0.4)	4–5

	Before training, I had…	**After the training, I had…**
**Relating to your understanding of what was involved in the role of a perinatal peer worker…**	*n* (%)	***n* (%)**
No understanding	1 (14.3%)	0 (0.0%)
Limited understanding	4 (57.1%)	0 (0.0%)
Reasonable understanding	2 (28.6%)	1 (14.3%)
Very high understanding	0 (0.0%)	6 (85.7%)

^a^ 1 = Strongly disagree, 2 = disagree, 3 = neither agree nor disagree, 4 = agree, 5 = strongly agree.

^b^ 1 = Completely disagree, 2 = somewhat disagree, 3 = neither agree nor disagree, 4 = somewhat agree, 5 = completely agree.

#### 4.3.2 Interview Findings

Interviews with Parent Peers further explored their feedback and recommendations relating to the training modules. Similar to the online survey responses, most Parent Peers reported that the online modules provided sufficient information and resources to prepare them for their role. However, several noted that additional time in the role may be necessary to identify knowledge gaps that emerge through practice. Only one participant expressed a need for additional content, suggesting the inclusion of ‘more real‐life scenarios or discussions we might come across during our work as a Parent Peer. This would then allow us to discuss as a group when we go through the module with the other Parent Peers.’

Parent Peers were aware of the importance of successful communication to their role, with active listening emerging as one of the most valued components of the training. Participants described the module on active listening as particularly impactful, with one noting, ‘The tools and techniques were good to unpack and made you really mindful of the way you communicate.’ One participant reflected on the impact of learning to embrace the silence and described a moment when this skill had helped her connect with a parent. ‘When I would have normally…filled it [the silence] in, she broke down and started to cry, and we made some really beautiful connection through that, which was really special.’

Aspects of the modules that encouraged self‐awareness and reflection were also highly valued. One Parent Peer declared that the reflective questions ‘allowed you to look inwards and decipher your own perspective and areas that you need to work on within yourself.’ Participants credited the modules with helping them become aware of how their questions, body language, and listening style could impact others, and either limit or expand a conversation. One Parent Peer noted that they now find themselves ‘talking and opening up more to others’ since completing the training. Having an awareness of their own biases and understanding the importance of body language were also identified as valuable learning outcomes.

The flexibility of completing modules at one's own pace was appreciated, although some participants expressed a desire to have ongoing access to the materials to review when necessary, supporting ongoing learning. One suggested a printed summary of key topics to enable continued reflection. The most commonly reported criticism of the modules was repetition, particularly within the Storytelling module, where participants felt that questions often elicited similar responses and that a mind map or brainstorming activity might have been a more effective format than writing a full narrative. Despite these concerns, participants generally found the modules to be relevant, well‐structured, and directly applicable to their work as Parent Peers. ‘I feel like I have had the most amount of training and onboarding for this role than any other role I have ever undertaken.’

The training also included face‐to‐face debriefing sessions held via teleconferencing software. These sessions were valued as they allowed Parent Peers to hear different points of view and discuss topics addressed in the modules and workbook, thereby supporting and reinforcing their learning. One Parent Peer described this form of open conversation as beneficial for her learning style, ‘it sort of opens the floor up for questions and to hear different perspectives’.

## 5 Discussion

The study described the co‐design, development, and early implementation of a Parent Peer programme to support parents living in rural and remote communities in Australia and explored Parent Peers' experiences of a hybrid training programme. Preliminary feedback was predominantly positive regarding the benefits of the Parent Peer model for supporting parents in rural and remote Australian communities. Interviews investigating Parent Peer and stakeholder experiences of the programme identified two themes: ‘Connection and Collaboration’ and ‘An Evolving Role’. The first theme came from stakeholders involved in the programme's design and development and highlighted the importance of bringing together a diverse range of perspectives and expertise for the co‐design process. The second theme, informed by all participants, reflected initial uncertainty about the nature of the role. There was a clear need for adaptability and flexibility until role boundaries and requirements were better understood, and to respond to emerging needs of the individual community. Parent Peers evaluated the hybrid training, providing predominantly positive responses, particularly relating to active listening, reflection, and ongoing discussion.

The development and implementation of the programme were supported by initial and ongoing collaboration between key stakeholder groups, with input and oversight from individuals with lived experience and childcare expertise. A growing body of research emphasises the importance of co‐design and co‐production, and a community‐led approach for the success, acceptability, relevance, and sustainability of community programmes [30, 31]. Similarly, the current study identified collaboration as a crucial aspect of the programme, described as vital to ensuring its relevance and effectiveness. The programme structure allowed each organisation to operate to their strengths; the research team led the research aspects of the programme, while OBF maintained a support role and maintained their relationships within the community. However, this means the research team may face barriers to recruitment and data collection throughout the project, as they lack the community relationships held by OBF and other organisational stakeholders. A future evaluation of the programme will investigate whether this separation of roles proves beneficial across all aspects of the project, or whether stakeholder roles should also remain flexible.

While described as evolving and context‐dependent, the Parent Peers' core responsibilities were defined as supporting, connecting, and advocating for rural parents. Stakeholders and Parent Peers acknowledged the difficulty of defining the role during these early phases of the project. This was not viewed as a concern but as a necessary aspect of the role, with all interviewees acknowledging the importance of allowing the role to evolve and adapt to the individual community's requirements. While previous peer support research has similarly found that less rigidly defined roles can be favourable for flexibility, a lack of role clarity was frequently described as a challenge associated with reduced support, insufficient training, and unclear professional boundaries [32, 33]. The fact that these challenges were less prominent in the current study may reflect the provision of comprehensive initial training, regular supervision, community support, and ongoing opportunities for debriefing and reflection. As the role continues to evolve, balancing flexibility with sufficient role clarity will be important to ensure Parent Peers feel adequately supported and prepared while maintaining a clear understanding of the boundaries and responsibilities of their role.

The programme's rural and remote setting is a significant factor driving the need for ongoing adaptability and reflexivity. Stakeholders spoke of recognising the unique challenges posed by geographic isolation, resource limitations, and cultural diversity, and the importance of being prepared for unexpected situations or changes. The ability to reflect upon experience, consider individual circumstances, and adapt the programme in response to these conditions was seen as essential to maintaining the programme's impact and relevance.

The evolving nature of the role also has implications for training. Participants anticipated that new training needs would emerge as Parent Peers encountered new and unanticipated challenges or scenarios. Through ongoing supervision and consultation, these needs could be fed back to training developers for regular updates of modules and other resources. This aligns with findings from previous studies that identified the need for comprehensive initial and ongoing training for peer workers, combined with regular, reflective supervision, particularly when supporting individuals with more complex needs than their own [32, 34].

Interviews with Parent Peers suggest that the hybrid training was beneficial and well‐received, particularly the aspects regarding effective listening and communication. Parent Peers reported feeling well‐equipped for the role thanks to the in‐depth training and onboarding processes provided by OBF. By offering flexible online learning modules and hardcopy workbooks to complement the live virtual debriefing sessions, the programme catered to a range of learning styles and schedules. The approach reduced the need for in‐person attendance and improved accessibility; an important consideration for those in rural and remote settings. The workbook allowed Parent Peers to revisit some of the materials at a later date. However, several Parent Peers felt that being able to access the modules themselves at a later time (either virtually or in booklet form) would provide a valuable resource to reinforce learning.

## 6 Implications and Future Directions

The study identified several implications for the future development and implementation of Parent Peer or similar support programmes in rural and remote Australian settings. Co‐design and programme development should be an iterative, ongoing process involving local community representatives, community organisations, people with lived experience, and topic experts to identify optimal strategies and characteristics that improve programme acceptability and sustainability. Mechanisms for obtaining ongoing community input after implementation would allow programmes and Parent Peers to better respond to the changing needs and unique circumstances of each community.

Training and debriefing should also be considered an ongoing component of programmes. Parent Peers described the benefit of regular debriefing sessions for reflecting, discussing challenges and ideas, and reinforcing learning. Hybrid or online training may be appropriate for ongoing or refresher learning, due to the geographical isolation in these areas. Continued access to training materials and activities, regular debriefing, and ongoing supervision may help Parent Peers prepare for challenging and complex situations, improve skills and knowledge, and reinforce previous learning. Training should be updated regularly to adapt to emerging learning needs as Parent Peers become more acquainted with their roles and as roles evolve.

While the programme may be transferable to other rural and remote communities in Australia, scalability will depend on local community populations and resources. Replicating the programme in other communities would not only require access to training materials and supervisors, but would also depend on the ability to recruit parents to the Parent Peer position, establish community partnerships, mobilise parents to attend Parent Peer‐affiliated groups, and access ongoing funding. Future implementation must therefore consider local recruitment potential and appropriate recruitment strategies, workforce retention, succession planning, supervision arrangements, and access to sustainable funding when determining whether a similar programme is suitable for specific communities.

## 7 Strengths and Limitations

A strength of the study was that most stakeholders involved in creating the programme took part in in‐depth interviews, allowing wide‐ranging insight into the development of the training and the programme, as well as reflections on performance and suggestions for improvement. All Parent Peers took part in interviews and completed online surveys. As the study was still in its early stages at the time of publication, we currently lack follow‐up data to evaluate the programme's outcomes and benefits. The small number of Parent Peers involved, and the limited number of study sites included may limit the programme's generalisability to other regional and remote areas of Australia.

## 8 Conclusion

This study describes the co‐design, development, and early implementation of a Parent Peer programme in rural and remote Queensland. Preliminary findings highlight the importance of ongoing stakeholder and community collaboration for the successful development, implementation, and sustainability of the programme. Feedback from Parent Peers identified priority training areas, such as active listening and resilience. The Parent Peer role needed to be flexible and adaptable to meet the unique and evolving needs of rural and remote communities. The Parent Peer model is a valuable resource that could be widely implemented in rural and remote communities across Australia to improve parent support and wellbeing. Additional longitudinal evaluation is required to assess Parent Peer, community and stakeholder perspectives on programme progress and value following a prolonged period of implementation. Longer‐term follow‐up is also needed to evaluate outcomes for parents in the community and programme sustainability. Implementing the model across additional sites, including more diverse communities, would help establish the model's transferability to different rural and remote settings.

## Author Contributions

**Kerri M. Gillespie:** investigation, formal analysis, writing – original draft preparation. **Megan McClenaghan:** formal analysis, writing – original draft preparation. **Nataya Branjerdporn:** conceptualisation, methodology, project administration, writing – review and editing. **Simon Jockel:** conceptualisation, methodology, project administration, writing – review and editing. **Grace Branjerdporn:** conceptualisation, methodology, supervision, writing – review and editing.

## Ethics Statement

The study was approved by the Mater Hospital Human Research Ethics Committee (ID: HREC/MML/103634). All participants provided written informed consent before taking part in any part of the research study.

## Conflicts of Interest

The authors declare no conflicts of interest.

## Use of Generative Artificial Intelligence

The authors confirm that no generative artificial intelligence tools were used in the writing or preparation of this manuscript.

## Data Availability

The data that support the findings of this study are available on request from the corresponding author. The data are not publicly available due to privacy or ethical restrictions.
